# Systematic Evaluation of Machine Learning and Deep Learning Models for IoT Malware Detection Across Ransomware, Rootkit, Spyware, Trojan, Botnet, Worm, Virus, and Keylogger

**DOI:** 10.3390/s26061750

**Published:** 2026-03-10

**Authors:** Mazdak Maghanaki, Soraya Keramati, F. Frank Chen, Mohammad Shahin

**Affiliations:** 1 Department of Mechanical, Aerospace, and Industrial Engineering, The University of Texas at San Antonio, San Antonio, TX 78249, USA; 2 Department of Mineral, Metallurgical, and Materials Engineering, Université Laval, Québec City, QC G1V 0A6, Canada; 3 Department of Industrial and Systems Engineering, University of Tennessee, Knoxville, TN 37996, USA

**Keywords:** IoT cybersecurity, malware detection, ransomware, trojan, rootkit, spyware, botnet, worm, virus, keylogger

## Abstract

**Highlights:**

**What are the main findings?**

- Ensemble tree-based machine learning models outperform deep learning architectures for feature-engineered IoT malware telemetry, with 7 of the top 10 models being ML-based.
- Optimal detection performance is malware-dependent, with gradient-boosted trees dominating most categories and tabular deep learning models excelling only in specific cases such as ransomware and virus detection.

**What are the implications of the main findings?**

- Gradient-boosted ensemble ML models provide the best accuracy–efficiency trade-off for practical IoT malware detection on commodity hardware.
- Model selection for IoT security should be data- and threat-aware, favoring classical ML for engineered telemetry and reserving deep learning for behavior-specific scenarios.

**Abstract:**

The rapid growth of Internet-of-Things (IoT) deployments has substantially expanded the attack surface of modern cyber–physical systems, making accurate and computationally feasible malware detection essential for enterprise and industrial environments. This study presents a large-scale, systematic comparison of 27 machine learning (ML) and 18 deep learning (DL) models for IoT malware detection across eight major malware categories: Trojan, Botnet, Ransomware, Rootkit, Worm, Spyware, Keylogger, and Virus. A realistic dataset was constructed using 50,000 executable samples collected from the Any.Run platform, including 8000 malware instances (1000 per class) and 42,000 benign samples. Each sample was executed in a sandbox to extract detailed static and behavioral telemetry. A targeted feature-selection pipeline reduced the feature space to 47 diagnostic features spanning static properties, behavioral indicators, process/file/registry activity, debug signals, and network telemetry, yielding a compact representation suitable for malware detection in IoT settings. Experimental results demonstrate that ensemble tree-based ML models consistently dominate performance on the engineered tabular feature set as 7 of the top 10 models are ML, with CatBoost and LightGBM achieving near-ceiling accuracy and low false-positive rates. Per-malware analysis further shows that optimal model choice depends on malware behavior. CatBoost is best for Trojan/Spyware, LightGBM for Botnet, XGBoost for Worm, Extra Trees for Rootkit, and Random Forest for Keylogger, while DL models are competitive only for specific categories, with TabNet performing best for Ransomware and FT-Transformer for Virus. In addition, an end-to-end computational time analysis across all 45 models reveals a clear efficiency advantage for boosted tree ensembles relative to most DL architectures, supporting deployment feasibility on commodity CPU hardware. Overall, the study provides actionable guidance for designing adaptive IoT malware detection frameworks, recommending gradient-boosted ensemble ML models as the primary deployment choice, with selective DL models only when category-specific gains justify additional computational cost.

## 1. Introduction

Malware or malicious software refers to any program or code intentionally designed to disrupt, damage, steal from, or gain unauthorized access to computing systems. Malware is not a single entity but a broad term encompassing multiple categories, including Trojans, botnets, ransomware, rootkits, worms, spyware, keyloggers, and viruses [1,2]. These malware types differ significantly in their infection vectors, persistence mechanisms, propagation strategies, and intended impact. Despite this diversity, malware is frequently and incorrectly referred to as viruses, whereas a virus represents only one specific subtype of malware that self-replicates by infecting host files. This oversimplification obscures the fact that different malware classes pose distinct threats and therefore require different detection and mitigation strategies, particularly in heterogeneous and resource-constrained environments [3]. Malware detection will be critical in the development of future Industry 6.0. Connected intelligent systems, robotics, and autonomous technologies rely on secure digital environments to function safely. In this context, strong malware detection combined with human–AI collaboration will help intelligent systems to identify threats faster and protect the advanced infrastructure that Industry 6.0 depends on [4].

**Figure 1:**
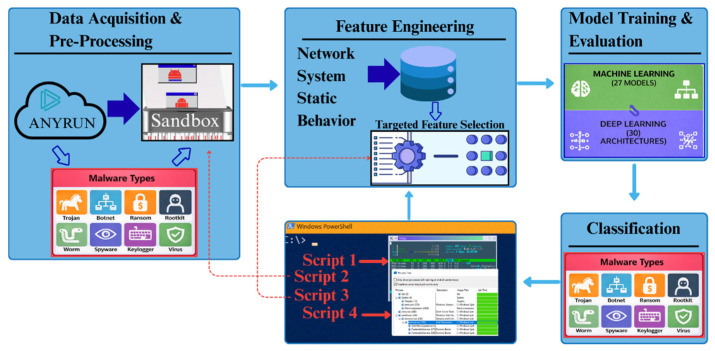
Conceptual workflow of the IoT malware detection process.

IoT systems consist of interconnected devices, sensors, gateways, communication networks, and cloud or edge-based analytics platforms that operate across multiple tightly coupled layers [5]. These layers from perception and data acquisition to networking, processing, and application are inherently interdependent, meaning that a compromise at one layer can propagate across the entire system. Over the past decade, IoT adoption has accelerated rapidly across diverse sectors, including manufacturing, healthcare, transportation, smart cities, and critical infrastructure [6,7]. However, IoT ecosystems are intrinsically vulnerable to cyber and malware attacks due to limited computational resources, heterogeneous hardware, long device lifecycles, and insufficient built-in security mechanisms. These weaknesses are particularly pronounced in lower-level IoT-covered enterprises, where cybersecurity infrastructure is often absent. IoT devices are continuously connected to the internet and a single compromised node can expose the broader system to cascading failures, data leakage, or operational disruption [8]. To address these risks, a wide range of malware detection techniques and cybersecurity defense mechanisms have been proposed, including signature-based detection, rule-based systems, heuristic analysis, behavior-based monitoring, and machine learning-driven classification. While traditional signature-based approaches are effective against known threats, they struggle to detect zero-day attacks, polymorphic malware, and evolving attack patterns commonly observed in IoT environments [9]. In contrast, feature-engineered detection approaches aim to identify deviations from normal system behavior, making them more adaptable and practical for dynamic and previously unseen threats [10]. Feature-based detection is particularly well-suited for IoT systems because it can operate on numerical telemetry, behavioral metrics, and network statistics without relying on explicit malware signatures [11]. A complete malware detection lifecycle typically involves data collection, feature extraction and selection, model training, evaluation, and deployment of an end-to-end framework conceptually illustrated in Figure 1.

This study addresses the need for a comprehensive and systematic malware detection framework tailored to IoT environments. A large-scale, real-world dataset comprising 50,000 samples was generated, including 8000 malware instances spanning eight predominant malware categories and 42,000 benign samples, enabling statistically reliable and generalizable analysis. Careful feature selection was performed to retain the most diagnostically relevant attributes while preserving the ability to distinguish not only malicious from benign behavior but also differences among malware types. The refined dataset was then evaluated using an extensive set of widely adopted machine learning and deep learning models to identify which models perform best for specific malware categories and to understand the underlying reasons for their performance. Based on these insights, this work provides practical recommendations to guide enterprises in selecting appropriate, data-driven defensive detection frameworks for securing IoT and IIoT systems.

In this work, the primary focus is on the comparative evaluation of machine learning and deep learning models for IoT malware detection. The objective is to assess the effectiveness of different detection models across multiple malware categories under standard dynamic analysis conditions. Therefore, the scope of the research is limited to the design and empirical evaluation of detection models. Aspects such as hardware-specific benchmarking on edge devices, adversarial malware evasion analysis, and long-term model maintenance considerations involve embedded-system optimization, adversarial machine learning frameworks, or operational lifecycle management, which are outside the scope of this paper. Similarly, modeling malware propagation dynamics or containment strategies in specialized environments falls outside the detection-centered objective of this research. Because the proposed approach relies on malware behavioral characteristics that are largely independent of specific IoT application scenarios, the framework is intentionally designed to be scenario-agnostic and focused on evaluating detection performance across multiple malware categories within a unified dataset. Defining these boundaries allows the work to provide a systematic and controlled evaluation of detection effectiveness while leaving deployment optimization, adversarial robustness, and environment-specific modeling as directions for future research.

## 2. Background

Malware detection has long been recognized as both a theoretically difficult and practically complex problem. From a theoretical perspective, malware detection is closely related to undecidable and NP-complete problems, particularly when malware exhibits self-modifying, polymorphic, or metamorphic behavior [12]. This implies that no algorithm can perfectly and universally detect all malware instances without false positives or false negatives [13]. In practice, this difficulty is amplified by the continuous evolution of malware techniques, including code obfuscation, encryption, packing, and multi-stage execution strategies designed explicitly to evade detection mechanisms [14]. Modern malware differs substantially from early, simplistic malicious programs. Contemporary malware frequently operates across multiple processes, leverages legitimate system components, dynamically alters its execution paths, and blends malicious actions with benign-looking behaviors. As a result, malware detection systems must balance accuracy, scalability, interpretability, and computational efficiency while operating under adversarial conditions [15]. These challenges have driven the development of a wide range of malware detection approaches, each addressing specific aspects of the problem space with distinct assumptions, strengths, and limitations. In Industry 6.0 environments, IoT forms the backbone of hyper-connected cyber–physical production systems, enabling continuous data exchange, distributed intelligence, and real-time orchestration of autonomous manufacturing processes. Consequently, strong detection mechanisms are critical to preserving system integrity and maintaining the resilience required for secure, self-adaptive industrial ecosystems [16].

Signature-based detection is one of the earliest and most widely deployed malware detection approaches, particularly in commercial antivirus systems [14]. This approach relies on identifying unique patterns referred to as signatures extracted from known malware samples. These signatures may consist of byte sequences, instruction patterns, string literals, hash values, or structural characteristics of executable files. During detection, incoming files are scanned and compared against a database of known signatures to determine whether they match previously identified malware [17]. The primary advantage of signature-based detection lies in its speed and efficiency. Since detection is essentially a pattern-matching operation, it imposes minimal computational overhead and can operate in real time. However, this approach is inherently limited to detecting known malware and fails against zero-day attacks, polymorphic malware, and obfuscated variants [18,19]. Even minor modifications to malware code can render signatures ineffective, forcing constant updates to signature databases and significant manual effort to maintain coverage [20]. Although enhancements such as automated signature generation and hybrid static–dynamic signatures have improved resilience to simple obfuscation techniques, signature-based detection remains fundamentally reactive and insufficient as a standalone defense mechanism in modern threat environments [21].

Behavior-based malware detection focuses on analyzing the runtime behavior of programs rather than their static code structure [22]. The underlying premise is that while malware code can change significantly across variants, its core malicious behaviors such as unauthorized file manipulation, suspicious network communication, privilege escalation, or persistence mechanisms remain relatively consistent [23]. Behavioral features are typically extracted through dynamic analysis techniques, including sandbox execution, system call monitoring, registry and file system tracking, network traffic analysis, and process interaction monitoring [24]. These features are then transformed into numerical representations and analyzed using statistical or machine learning classifiers to distinguish malicious from benign behavior [25]. Behavior-based approaches are particularly effective against unknown and obfuscated malware and can detect entire malware families based on shared behavioral traits [26]. However, they introduce new challenges, including higher computational cost, dependence on accurate execution environments, and susceptibility to evasion techniques such as environment-aware malware that suppresses malicious behavior when executed in sandboxes [27]. Additionally, benign applications may exhibit behaviors similar to malware under certain conditions, leading to increased false positive rates [28].

Heuristic-based detection represents an intermediate approach that combines expert-defined rules, behavioral indicators, and machine learning techniques to identify suspicious activity [29]. Rather than relying on exact signatures, heuristic systems evaluate whether a program exhibits patterns commonly associated with malicious behavior, such as abnormal API usage, suspicious instruction sequences, or unusual control flow structures [30,31,32]. Heuristic detection systems can identify previously unseen malware and are more flexible than pure signature-based methods. They often leverage features such as opcode distributions, control flow graphs, API call sequences, and hybrid static–dynamic representations [33]. However, heuristic approaches typically require extensive tuning, handcrafted rules, and careful threshold selection, making them prone to false positives and difficult to scale. As malware grows more complex and adaptive, heuristic-based detection struggles to maintain accuracy without becoming overly restrictive or computationally expensive [34].

Model checking-based malware detection applies formal verification techniques to identify malicious behaviors encoded as logical specifications [35]. In this approach, malware behaviors are represented using temporal logic formulas that describe sequences of system events or state transitions indicative of malicious intent [35,36]. Executable programs are converted into abstract models such as control flow graphs or state machines which are then verified against these specifications [37]. This approach offers strong theoretical guarantees and can detect malware variants using a single behavioral specification. It is particularly effective against obfuscation and polymorphism, as detection relies on semantic behavior rather than syntactic structure [38]. However, model checking is computationally intensive, difficult to automate fully, and often requires manual specification of malicious behaviors. As a result, model checking-based detection is best suited for targeted analysis and forensic investigation rather than large-scale real-time deployment [39,40].

Machine learning has become a cornerstone of modern malware detection due to its ability to learn complex patterns from large datasets [41]. Traditional machine learning models including decision trees, support vector machines, k-nearest neighbors, random forests, and probabilistic classifiers have been widely applied using both static and dynamic features. The effectiveness of machine learning-based detection depends heavily on feature quality and selection. High-dimensional feature spaces can lead to overfitting, increased training time, and reduced interpretability [42]. Consequently, feature selection plays a critical role in identifying the most discriminative attributes while maintaining generalization across malware families. While classical machine learning models offer good performance and interpretability, they often struggle to capture highly nonlinear relationships present in complex malware behaviors [25,43,44,45,46,47,48,49,50].

Deep learning extends machine learning by enabling hierarchical feature learning through multi-layer neural networks [51]. Deep models can automatically extract high-level representations from raw inputs such as byte sequences, system call traces, or API call graphs, reducing reliance on manual feature engineering. Deep learning-based approaches have demonstrated strong performance in large-scale malware classification tasks and can handle high-dimensional data effectively [52]. Architectures such as deep neural networks, convolutional neural networks, and recurrent neural networks have been applied to both static and dynamic malware analysis [53]. Despite these advantages, deep learning models are computationally expensive, require large labeled datasets, and are vulnerable to adversarial evasion attacks. Additionally, increasing model depth does not always yield improved performance, and interpretability remains a significant challenge in security-critical applications [14,15,54,55,56,57,58,59,60].

Cloud-based frameworks leverage centralized computational resources and large-scale malware databases to enhance detection accuracy and scalability [61,62]. In this model, lightweight clients collect features or upload samples to cloud servers, where intensive analysis and classification are performed [63,64]. Cloud-based approaches enable continuous updates, cross-device intelligence sharing, and the application of complex models unsuitable for resource-constrained devices [65,66,67]. However, they introduce concerns related to privacy, latency, bandwidth consumption, and dependence on reliable network connectivity. These trade-offs are particularly important in IoT environments, where real-time detection and low communication overhead are critical [68,69].

The rapid expansion of mobile and Internet-of-Things ecosystems has introduced significant challenges for effective malware detection [70]. IoT devices are typically resource-constrained, highly heterogeneous, and often deployed with minimal security configurations, making them attractive targets for attackers [71]. Malware targeting IoT environments frequently exploits weak authentication mechanisms, unpatched firmware, and insecure communication protocols [72]. Consequently, IoT-focused malware detection approaches place strong emphasis on lightweight feature extraction, energy-efficient monitoring, and scalable detection architectures [72]. Detection techniques include system call monitoring, network traffic analysis, energy consumption profiling, and hybrid cloud–edge frameworks [70]. While promising, IoT malware detection remains in an early stage, with limited datasets, evolving threat models, and ongoing challenges in scalability and generalization [73].

In parallel with the development of malware detection techniques, several benchmark datasets have been introduced and widely adopted for evaluating IoT-focused malware detection systems. Among the most frequently cited are TON-IoT, Bot-IoT, IoT-23, CIC-IoT-2023, and N-BaIoT [74,75,76,77]. While these datasets have played an important role in advancing research, they exhibit notable limitations that reduce their suitability for rigorous machine learning and deep learning evaluation. Many include features such as hash values, file identifiers, timestamps, or dataset-specific metadata that do not reflect intrinsic malicious behavior and offer little meaningful learning signal for AI models [78]. In several cases, samples are labeled only at a coarse level as “malware” or “benign,” without distinguishing between different malware categories, despite the fact that Trojans, ransomware, botnets, and spyware exhibit fundamentally different behavioral and operational characteristics [79,80]. This aggregation of heterogeneous malware into a single label can lead to misleading performance metrics and models that fail to generalize beyond the dataset [81]. Furthermore, some datasets rely on synthetic traffic generation or outdated attack scenarios, limiting their relevance to contemporary IoT threat landscapes [82]. In contrast, this study addresses these shortcomings by generating an up-to-date, real-world IoT malware dataset derived from sandboxed execution reports, consisting exclusively of meaningful numerical behavioral and static features. The dataset explicitly represents eight predominant malware types alongside benign samples and undergoes careful feature selection to eliminate non-informative attributes. The resulting dataset provides a clean, realistic, and easy-to-use foundation for systematic AI-based malware analysis, enabling more reliable performance evaluation and practical deployment of malware detection frameworks. To further contextualize the complexity of IoT malware detection and to highlight the multidimensional nature of malicious behavior, Table 1 provides a structured overview of representative malware behaviors, attack objectives, IoT architectural layers, delivery vectors, traffic characteristics, and underlying security weaknesses. Rather than enumerating specific attacks in isolation, the table organizes these behaviors in terms of their observable anomalies and system-level manifestations, emphasizing how diverse malware strategies translate into measurable deviations across network, device, firmware, and application layers. This abstraction is particularly important for detection, where models are trained to recognize deviations from normal operation rather than predefined attack signatures, by mapping attack behaviors to detection-relevant dimensions such as traffic intensity, resource consumption, access violations, and data handling irregularities. Table 1 illustrates the rationale for focusing on carefully selected numerical features and multi-class labeling. This conceptual framework directly motivates the dataset design and feature selection strategy adopted in this study and clarifies how different malware types can be distinguished based on their anomalous impact on IoT systems rather than superficial identifiers or dataset-specific artifacts.

## 3. Dataset

A comprehensive and well-constructed dataset is a fundamental prerequisite for reliable machine learning and deep learning-based malware detection, particularly in IoT environments where system heterogeneity and attack diversity are pronounced [83]. From a learning perspective, an effective dataset must satisfy several critical criteria: sufficient scale to capture real-world variability, diversity across malware categories, realistic representation of malicious and benign samples, and clean, consistent labeling that enables reproducible analysis [84,85,86]. Datasets that fail to meet these criteria often lead to biased learning, inflated performance metrics, or limited generalizability in practical deployments [87]. In this study, a dataset comprising 50,000 samples was constructed to reflect a realistic IoT-enabled enterprise environment, such as factories, facilities, and industrial systems instrumented with interconnected sensors and devices. Of these samples, 8000 correspond to malware instances, evenly distributed across eight predominant malware categories, while the remaining samples represent benign activity. This scale was deliberately selected to strike a balance; in other words, it is sufficiently large to capture diverse operational and attack scenarios encountered in real-world IoT deployments, yet not excessively large in a way that would introduce unnecessary computational overhead or diminishing analytical returns [88]. As a result, the dataset supports rigorous evaluation of detection models while remaining practical for systematic experimentation and comparative analysis. The dataset generation process was designed as a structured, multi-stage pipeline to ensure data quality, relevance, and consistency. First, representative samples were collected from real-world malware repositories, capturing both malicious and benign ones relevant to IoT and enterprise environments. Second, all samples were subjected to sandboxed execution to extract detailed static and behavioral reports under controlled conditions. This sandboxing stage enabled the capture of rich numerical telemetry describing system interactions, resource usage, and network activity without relying on superficial identifiers. Finally, a targeted feature selection process was applied to remove non-informative, redundant, or misleading attributes, retaining only those features most relevant to malware detection and differentiation.

### 3.1. Sample Collection

**Figure 2:**
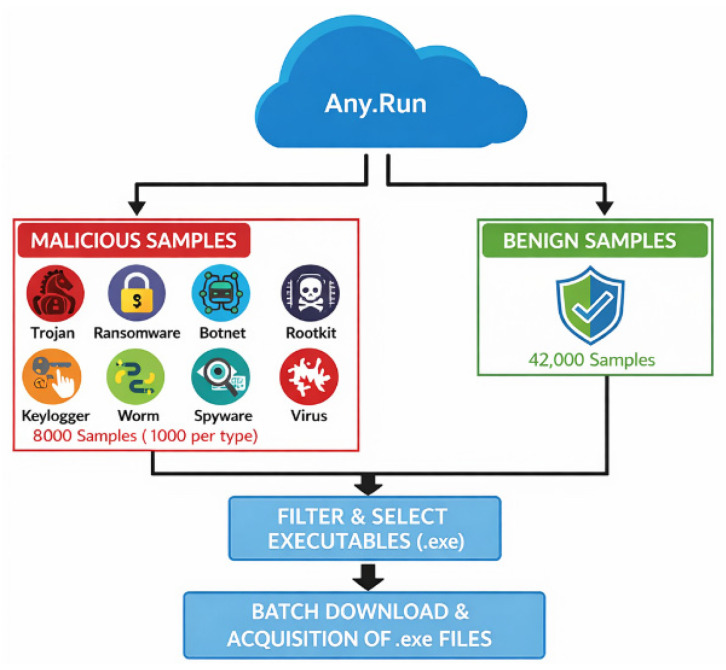
Sample collection workflow with executable filtering.

All samples used in this study were collected via the Any.Run online malware analysis platform, a continuously updated repository that hosts more than two million malware samples contributed and maintained by a global user community [89]. Because new samples are added at any time, the repository reflects current and real-world threat landscapes rather than synthetic or outdated attack scenarios. This ensures that the collected data are representative of contemporary malware observed in operational environments [90]. The sample collection process is straightforward and scalable. Any.Run provides an integrated search engine that allows users to filter and select samples based on malware type, enabling targeted acquisition across multiple threat categories. In addition, the platform supports batch downloading, which allows large bundles of samples to be retrieved efficiently in a single operation. This capability was essential for assembling a dataset of sufficient size while maintaining consistency across malware classes. For uniformity and efficiency in downstream analysis, the collected samples were restricted to executable (.exe) files. Although IoT environments may be affected by malware delivered through other formats such as scripts, links, or malicious media, this study focuses on executable files for two main reasons. First, executable files enable consistent sandbox execution and reliable dynamic feature extraction. Second, publicly available data sources containing non-executable IoT malware formats are limited and insufficient for building a balanced dataset. Both malicious and benign data were obtained through the same collection pipeline to ensure consistency in sample handling and processing. The overall workflow of the data collection stage, from repository query to executable acquisition, is illustrated in Figure 2.

### 3.2. Sandboxing and Report Generation

Following sample collection, all samples were analyzed using the Any.Run sandboxing environment [89]. Sandboxing is a critical step in malware dataset generation, as it enables controlled execution of potentially malicious code in an isolated virtual environment, thereby eliminating any risk to local systems or institutional infrastructure [91,92]. By executing malware within a sandbox, it is possible to safely observe runtime behavior, system interactions, and network activity that would otherwise be inaccessible through static analysis alone [93]. Any.Run provides a robust and user-friendly sandboxing workflow, particularly in its professional mode, which allows users to upload and execute multiple samples simultaneously. This capability was essential for efficiently processing large bundles of executable files and ensuring consistency across analyses. During execution, each sample runs inside a fully instrumented virtual machine that monitors file system activity, process creation, memory usage, registry modifications, API calls, and network communications in real time. Importantly, the platform supports extended execution times, which is crucial for malware families that exhibit dormant or delayed activation behavior. By allowing samples to execute beyond their initial launch, the sandbox increases the likelihood of triggering malicious routines that are intentionally designed to evade short-lived analysis [94].

**Figure 3:**
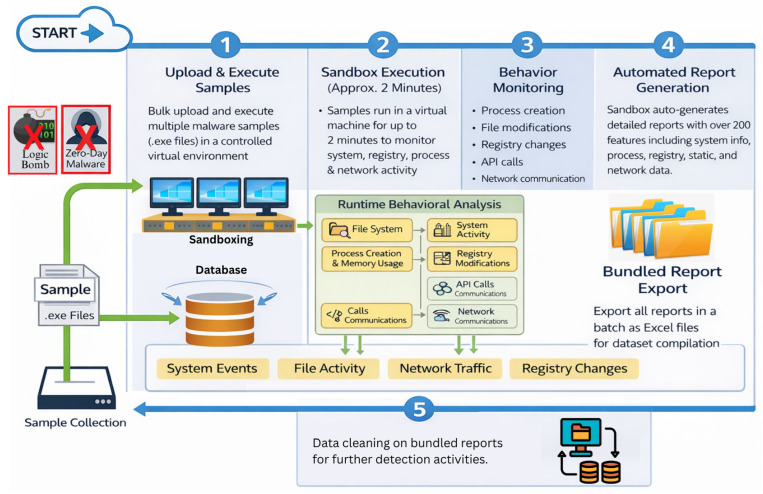
Sandbox execution and automated report generation workflow.

In this study, an execution window of approximately two minutes was selected for each sample. This choice reflects a practical trade-off between coverage and scalability. While certain classes of malware particularly zero-day or highly evasive threats may require longer or condition-specific activation periods, it is widely observed that the majority of common malware families initiate detectable malicious activity shortly after execution [95]. We therefore assume that this execution window captures the dominant behavioral signatures of most malware samples included in the dataset. Malware that remains inactive beyond this window is acknowledged as a limitation inherent to dynamic analysis and is discussed later in the paper. The most critical outcome of the sandboxing stage is automated report generation. For each executed sample, Any.Run produces a comprehensive analysis report that aggregates information across multiple domains, including system-level events, static file properties, process and memory activity, registry operations, network traffic, and high-level behavioral indicators. Each report contains more than 200 distinct attributes with corresponding numerical or categorical values, providing a rich and structured representation of sample activity. Also, reports are generated under identical sandbox configurations. A key advantage of this platform is its ability to export reports in batch mode and in spreadsheet-compatible formats, which significantly simplifies downstream dataset compilation and preprocessing. The overall sandboxing and report generation workflow is illustrated in Figure 3.

### 3.3. Feature Selection

Following sandbox execution and automated report generation, each analyzed sample produced a detailed report containing more than 200 attributes spanning static properties, runtime behavior, system interactions, and network activity. While such richness provides comprehensive visibility into sample execution, directly using all available attributes for machine learning and deep learning analysis can be counterproductive. High-dimensional feature spaces increase computational cost, introduce redundancy, amplify noise, and may cause models to learn dataset-specific artifacts rather than generalizable malicious patterns [96]. To address this challenge, a targeted feature selection strategy was applied based on prior knowledge of malware detection techniques discussed in Section 2. The objective was to identify a compact yet expressive subset of diagnostic features that (i) capture meaningful deviations from benign behavior, (ii) enable reliable discrimination between malware and benign samples, and (iii) preserve sufficient granularity to distinguish between different malware categories. Importantly, all features were extracted post-execution only, and no execution-specific identifiers (sample ID, run ID, file hash, timestamps) were used. Emphasis was placed on selecting numerical and categorical features that are consistently observable across samples and directly relevant to feature-based detection. The final feature set consists of 47 carefully selected features, grouped into eight information categories: static information, behavioral indicators, configuration information, process activity, file activity, registry activity, debug information, and network information. Each category contributes a complementary view of malicious execution, enabling models to learn multi-dimensional representations of malware activity.

Static information provides an initial characterization of malware and offers insight into potential obfuscation and packing strategies commonly used by malware [97]. Features such as file size and PE file entropy help identify abnormal compression or encryption patterns, while the compiler or packer indication derived from TRiD analysis can reveal attempts to evade static inspection [98]. The imported library count further reflects functional complexity and potential misuse of system-level APIs. Together, these static features establish a baseline for distinguishing suspicious binaries from benign software [99]. Behavioral information plays a central role in malware detection by capturing how malware acts during runtime. Indicators such as a suspicious behavior attempt, persistence behavior indicator, and privilege escalation attempt flag summarize high-risk actions that deviate from normal application behavior [100]. Additional behavioral features, including anti-analysis or evasion behavior, process injection behavior, self-modifying code behavior, and delayed execution behavior, are particularly useful for identifying malware designed to bypass sandbox environments [101]. An unauthorized system interaction flag further captures anomalous access to protected system resources. These features collectively encode high-level intent rather than superficial characteristics. Configuration-related features capture how executables interact with their execution environment [102]. The execution environment type and use of elevated privileges indicate whether malware attempts to exploit administrative contexts, while a sandbox detection value reflects efforts to identify and evade analysis environments. Environment variable modification and system configuration access attempts are often associated with persistence mechanisms or system reconnaissance, making them valuable signals for detection [103]. Process activity features describe how malware interacts with the operating system at runtime. The number of processes created and suspicious parent–child process relationships help identify abnormal process spawning behavior [104]. Process memory usage peak and process injection attempts are indicative of code injection or stealth execution [105]. Additional features such as process termination anomalies, hidden or background process creation, command-line argument entropy, and API call frequency related to process activity capture subtle deviations in execution patterns that are difficult to conceal and highly informative for classification [106]. File activity features capture unauthorized or excessive interactions with the file system. The number of files created, modified, or deleted reflects destructive or persistent behavior, while access to system directories and creation of hidden files often indicate attempts to manipulate protected resources [107]. File write frequency further quantifies abnormal file system usage that may be associated with payload deployment or data manipulation [108]. Registry activity features are particularly important for detecting persistence and configuration tampering [109]. The number of registry keys created or modified, access to autorun registry entries, and a registry persistence value directly reflect attempts to survive reboots [110]. Registry access frequency and unauthorized registry hive access further capture anomalous interactions with critical system configuration stores [111]. Debug-related features provide insight into malware awareness of analysis environments. Indicators such as debugger detection, use of anti-debugging APIs, exception handling anomalies, and timing-based evasion values are commonly associated with advanced malware families that attempt to disrupt dynamic analysis and conceal malicious intent [112,113]. Network information features describe external communication patterns, which are essential for detecting botnets, command-and-control activity, and data exfiltration [114]. The number of outbound connections and destination IP diversity capture communication breadth, while unusual port usage and network packet rate highlight deviations from normal traffic patterns [115]. Data volume transmitted provides a quantitative measure of potential exfiltration or flooding behavior, and an encrypted communication value reflects attempts to conceal malicious traffic [116]. Table 2 summarizes the complete list of selected features used for dataset construction and subsequent analysis.

## 4. Methodology

To systematically evaluate the effectiveness of artificial intelligence techniques for IoT malware detection, this study employs a broad spectrum of machine learning and deep learning models applied to the generated dataset described in Section 3. The selected models span linear, nonlinear, ensemble-based, probabilistic, distance-based, and neural architectures, enabling a comprehensive comparison across different learning paradigms. For all models, a fixed 80/20 train–test split was applied with class proportions preserved to ensure consistent and reproducible evaluation and all models are trained and evaluated on the same feature-selected dataset. To verify that model performance was not driven by execution artifacts or feature leakage, validation analyses were conducted. Feature variance analysis confirmed diversity across behavioral and network features, indicating non-homogeneous execution outcomes across samples.

### 4.1. Machine Learning Models

Traditional machine learning models remain widely used in cybersecurity due to their interpretability, computational efficiency, and strong performance on structured numerical data. In this study, 27 machine learning models are evaluated, covering both supervised classification and unsupervised detection approaches. Logistic Regression serves as a baseline linear classifier that models the probability of class membership and performs well when feature relationships are approximately linear [117,118]. Gaussian and Multinomial Naïve Bayes classifiers rely on probabilistic assumptions and conditional independence between features, making them computationally efficient and effective for high-dimensional data with relatively simple distributions [119,120]. Linear Discriminant Analysis and Quadratic Discriminant Analysis model class separation using Gaussian assumptions, with QDA providing additional flexibility when class covariances differ [121,122]. The Ridge Classifier introduces regularization to linear classification, improving robustness against multicollinearity [123,124]. Support Vector Machines with linear and radial basis function kernels are employed to capture both linear and nonlinear decision boundaries, with the RBF variant being particularly effective for complex feature interactions [125]. The k-Nearest Neighbors algorithm provides a distance-based perspective, performing well when local similarity patterns exist in the feature space [126]. Tree-based models, including Decision Tree, Random Forest, and Extra Trees, are used to capture nonlinear feature interactions and hierarchical decision structures [127]. Random Forest and Extra Trees improve generalization through ensemble learning and randomized splits [128,129]. Gradient Boosting, AdaBoost, XGBoost [130], LightGBM, and CatBoost represent boosting-based ensemble methods that iteratively refine weak learners, often achieving strong performance on tabular data and handling feature importance effectively [131]. Online and margin-based learners such as the SGD Classifier, Passive-Aggressive Classifier, and Perceptron are included due to their scalability and suitability for large datasets [132]. Extreme Learning Machine offers fast training by randomly initializing hidden layers, making it attractive for rapid experimentation [133]. Unsupervised and semi-supervised anomaly detection models are also incorporated, including One-Class SVM, Isolation Forest, Local Outlier Factor, and Elliptic Envelope, which are particularly relevant for scenarios where labeled malware samples may be scarce or evolving [111,112]. Bayesian Network models probabilistic dependencies among features, providing interpretability, while RuleFit combines rule-based learning with linear models to balance transparency and predictive power [134,135].

### 4.2. Deep Learning Models

Deep learning models are increasingly adopted for malware detection due to their ability to learn hierarchical and nonlinear representations from complex data. In this work, 18 deep learning architectures are evaluated to assess their suitability for numerical IoT malware data and anomaly detection tasks. Feedforward architectures such as the Multilayer Perceptron and Deep Neural Network form the foundation for nonlinear modeling, while Residual MLP introduces skip connections to stabilize deeper networks [136,137]. One-dimensional Convolutional Neural Networks are used to capture local and temporal feature patterns, even in non-image numerical data [138]. Sequential models are applied to capture temporal dependencies in execution and network-related features [139]. Bidirectional variants further enhance contextual learning by processing sequences in both forward and backward directions [140]. Autoencoder-based models, including Autoencoder, Stacked Autoencoder, Variational Autoencoder, and Denoising Autoencoder, are employed for anomaly detection by learning compact representations of benign behavior and identifying deviations. Transformer-based architectures, including Transformer Encoder and Temporal Transformer, leverage self-attention mechanisms to model global feature dependencies. TabNet and FT-Transformer are specifically designed for tabular data, providing interpretability and strong performance on structured numerical datasets. Generative models, including the Generative Adversarial Network and CTGAN, are considered for their ability to model complex data distributions and support anomaly detection or data augmentation. Deep Belief Network and Deep Boltzmann Machine represent probabilistic deep architectures capable of learning hierarchical feature distributions [141]. Capsule Network introduces vector-based representations to preserve spatial and relational information, while Attention-based Neural Networks enhance focus on salient features [142]. Finally, Multi-task Neural Networks are included to explore joint learning across multiple malware categories and detection objectives [143,144]. Table 3 summarizes all machine learning and deep learning models implemented in this study. The table enumerates the full set of 45 models. This comprehensive model coverage enables an objective assessment of how different learning paradigms perform under identical data conditions, facilitating robust conclusions regarding model suitability for IoT malware detection across diverse attack scenarios.

## 5. Results and Discussion

**Figure 4:**
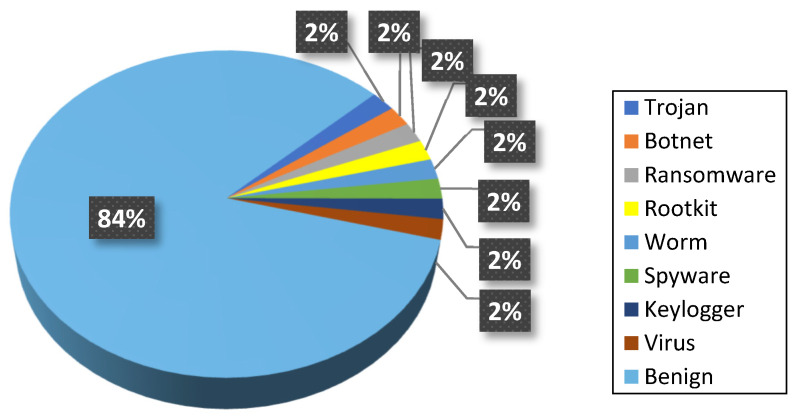
Distribution of benign and malware samples in the dataset.

The generated dataset consists of 50,000 executable files, including 8000 malware samples evenly distributed across eight predominant malware categories—Trojan, Botnet, Ransomware, Rootkit, Worm, Spyware, Keylogger, and Virus—alongside 42,000 benign samples. In all experiments, a fixed train–test split was used to ensure consistency and reproducibility across model evaluations. Specifically, 80% of the dataset was allocated for training and 20% for testing, with class proportions preserved across both subsets. A temporal split was not applied because the dataset does not reflect a continuous or chronological deployment environment. This composition reflects practical enterprise and industrial IoT environments, where benign activity dominates but diverse malware threats coexist. The realistic representation of malware classes, combined with a significantly larger benign set, provides a realistic and challenging foundation for detection and multi-class classification tasks. The overall distribution of malware and benign samples within the dataset is illustrated in Figure 4, which highlights both the dominance of benign activity and the equal representation of malware types. Such a distribution is essential for evaluating detection performance under realistic operating conditions and avoiding bias toward any single malware family.

**Figure 5:**
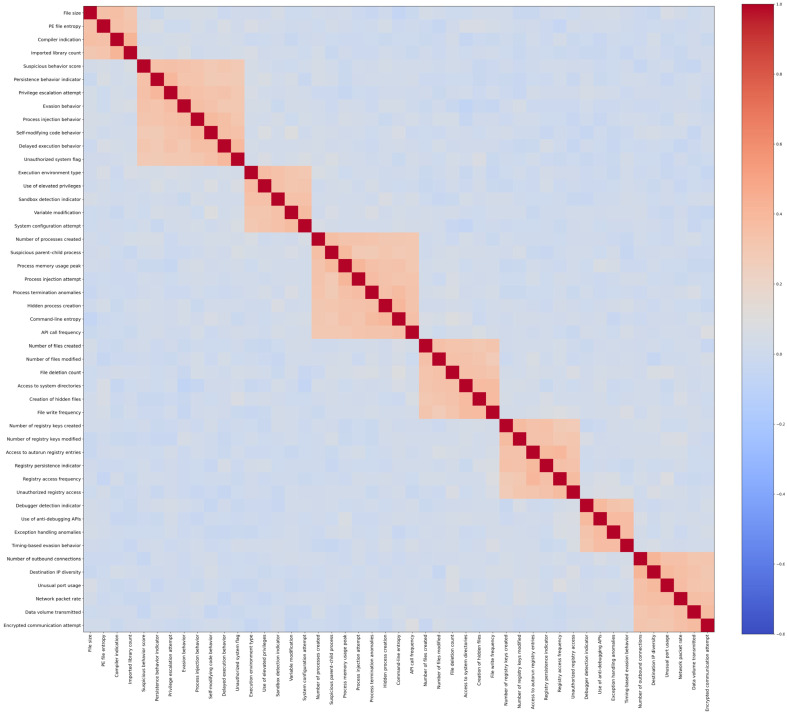
Correlation matrix of the 47 selected diagnostic features.

To further validate the suitability of the selected feature set, a correlation analysis was conducted on the 47 diagnostic features described in Section 3.3. Feature redundancy and excessive correlation can negatively impact learning by introducing noise, increasing model complexity, and reducing interpretability. Therefore, a correlation matrix was computed to examine pairwise relationships among all features, as shown in Figure 5. The analysis reveals no highly positive correlations that would indicate redundant or near-duplicate features. Instead, the majority of feature pairs exhibit low to moderate correlation, suggesting that each feature contributes complementary information. This confirms that the feature selection process successfully retained informative attributes while avoiding unnecessary overlap, thereby enhancing the robustness and generalizability of subsequent machine learning and deep learning models.

**Figure 6:**
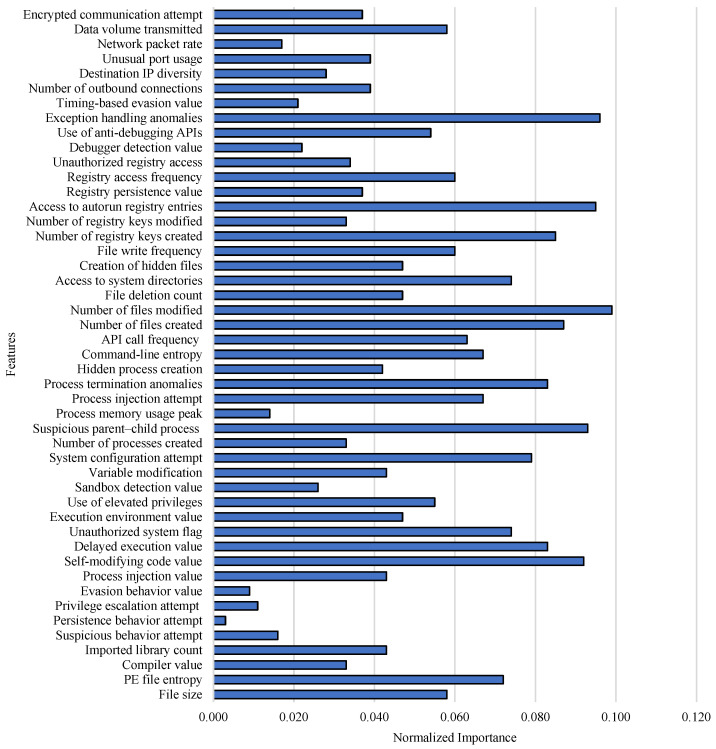
Feature-importance analysis of selected features.

To ensure a fair and reproducible comparison among the evaluated algorithms, all ML and DL models were implemented using standardized parameter settings rather than extensive per-model hyperparameter optimization. Performing independent optimization for each of the 45 models would introduce tuning bias and make comparative evaluation inconsistent, as different algorithms respond differently to search strategies and computational budgets. Therefore, widely accepted default configurations and commonly reported parameter ranges from prior studies were adopted. For ML models, parameters such as tree depth, number of estimators, and regularization settings were selected based on standard library recommendations. For DL models, consistent training conditions were used, including identical training–testing splits, learning rate initialization, batch size, number of epochs, and optimizer (Adam). This approach is frequently used in comparative machine learning studies to evaluate relative model behavior under uniform experimental conditions, ensuring that performance differences arise from model capability rather than unequal tuning effort. To further strengthen the feature selection process, a feature-importance analysis was performed (Figure 6). The analysis revealed that the number of files modified, exception-handling anomalies, and access to the autorun registry entry are the three most significant features for malware detection in this study.

To evaluate detection effectiveness under consistent experimental conditions, multiple performance metrics were computed for each model, including training accuracy, test accuracy, precision, recall, F1-score, and false positive rate (FPR). Training accuracy is reported to indicate model fit, while test accuracy reflects generalization to unseen samples. Precision and recall quantify complementary error characteristics: precision captures how reliably predicted malware is truly malicious which is important for operational trust, whereas recall reflects the ability to detect malicious samples without misses. The F1-score summarizes the precision–recall trade-off, and FPR is emphasized because false alarms are operationally expensive in IoT-enabled enterprises and can reduce confidence in a deployed system. Because the dataset supports both malware-versus-benign detection and malware-type discrimination, model performance was analyzed in two complementary ways. First, all 45 models were benchmarked using the same feature-selected dataset to ensure fair comparison across learning paradigms. Second, results were examined per malware type to identify which models best detect specific threat classes. This per-class view is important because different malware families produce distinct statistical patterns in the selected features. Accordingly, the best-performing models can vary by malware category, and a single “global best” model may not be optimal across all incidents. Table 4 provides a comprehensive comparison of 45 models (27 ML and 18 DL) evaluated on the same 47-feature dataset using Train Accuracy, Accuracy, Precision, Recall, F1, and FPR. Several high-level conclusions emerge immediately. Importantly, the performance comparison for all models was done in a binary setup, malware versus benign.

**Figure 7:**
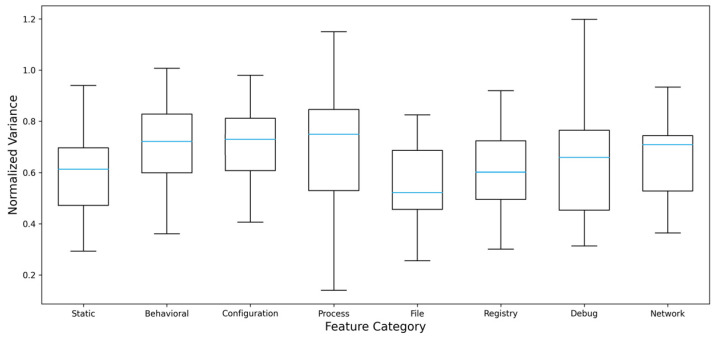
Normalized feature variance across extracted features.

To exclude the possibility that model performances were driven by execution artifacts, feature leakage, or overly homogeneous sandbox behavior, a feature variance analysis was conducted. As shown in Figure 7, normalized feature variance exhibits dispersion across multiple feature groups. This indicates that execution traces did not collapse to a uniform or deterministic profile despite the use of a fixed sandbox configuration.

**Figure 8:**
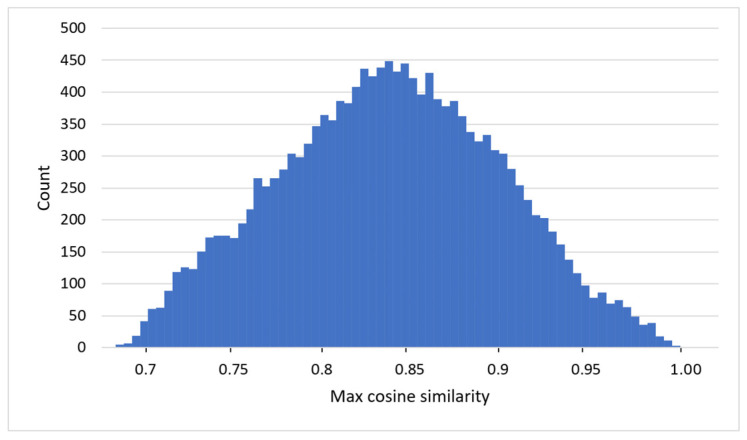
Train–test near-duplicate leakage audit.

To verify that the reported detection performance is not inflated by duplicate or near-duplicate samples across the training and test sets, a leakage audit was performed. For each test sample, the maximum cosine similarity between its standardized 47-feature vector and all training samples was computed using training-set statistics only. High-similarity values would indicate potential duplicate or near-duplicate leakage arising from repeated uploads, minor packing variants, or closely related malware builds. As shown in Figure 8, the distribution of maximum test-to-train similarity values is broad and does not exhibit a concentration near unity. Only a negligible fraction of test samples approach conservative near-duplicate thresholds (0.99 and 0.995), indicating that test instances are not near replicas of training data. This analysis confirms that the strong classification performance reported in Table 4 reflects genuine generalization rather than memorization driven by duplicate or near-duplicate leakage.

Across the 45 evaluated models, the top-10 is dominated by gradient-boosted tree ensembles (CatBoost, LightGBM, XGBoost, Gradient Boosting, Extra Trees, Random Forest) with only tabular-specialized deep architectures (TabNet, FT-Transformer, Transformer Encoder) consistently reaching comparable accuracy. This outcome is technically expected given the dataset’s representation: the input is a compact 47-dimensional numerical feature vector distilled from static and behavioral sandbox reports, which defines a prototypical tabular learning regime. GBDT ensembles are statistically efficient on tabular data and excel at modeling high-order, non-additive feature interactions through hierarchical thresholding, while remaining robust to heterogeneous feature scales, heavy-tailed count distributions. In contrast, many deep architectures in the benchmark impose inductive biases aligned to spatial, temporal, or relational structure that is not explicitly present in a flat feature vector, which limits their ability to exploit the underlying signal without a representation redesign (e.g., event sequences or behavior graphs). The strongest DL results are therefore achieved by architectures explicitly designed to learn feature interaction structure in tabular settings (TabNet/FT-Transformer), narrowing—but not surpassing—the performance gap to the best GBDTs. Overall, these results indicate that for engineered numerical telemetry features, modern tree boosting provides the most reliable accuracy–complexity trade-off, while tabular DL becomes competitive primarily when its interaction-learning mechanisms (feature-wise embeddings/attention) match the statistical structure of the data. A secondary analysis evaluates the models’ efficacy in identifying specific malware families. Results discussed regarding ‘Best Model per Category’ utilize a one-vs.-rest evaluation framework to determine category-specific deployment recommendations (Table 5).

**Figure 9:**
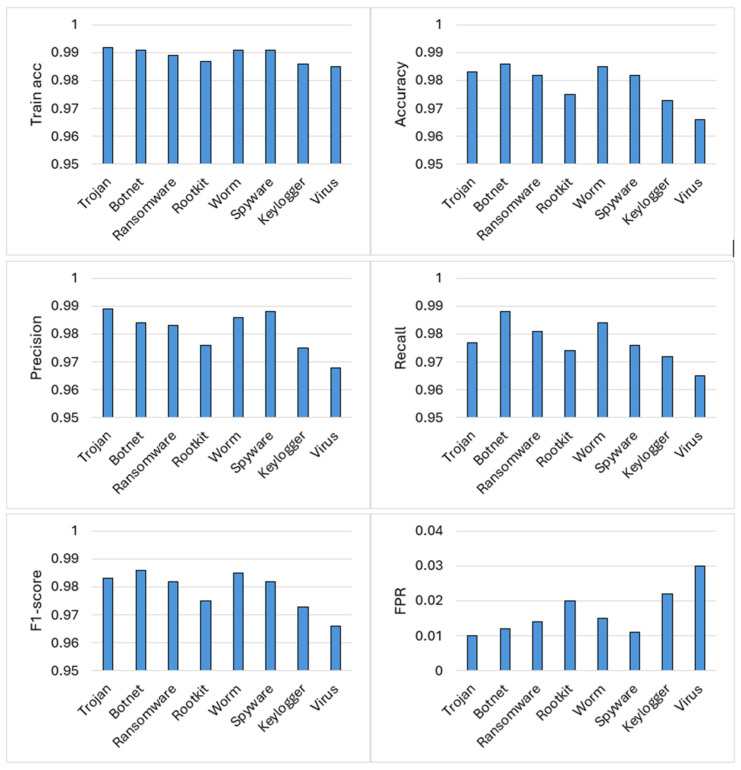
Performance metrics of the best-performing model for each malware category.

Unlike aggregate evaluations, this per-malware analysis reveals that detection performance is strongly controlled by the interaction between malware behavioral characteristics, feature-space structure, and the inductive bias of the learning algorithm. A consistent trend emerges in which classical machine learning models particularly gradient-boosted and ensemble tree methods outperform most deep learning architectures, with deep models becoming competitive only when their architectural assumptions closely align with the underlying data representation. For Trojan detection, CatBoost achieves the highest performance, as shown in Figure 9. CatBoost’s ordered boosting framework and strong regularization enable it to capture such high-order feature interactions while maintaining robust generalization under class imbalance, resulting in both high accuracy and low false-positive rates. Botnet activity is dominated by network-centric behaviors such as periodic beaconing, repetitive command-and-control communication, and burst-like connection patterns. Although such behavior is inherently temporal, the sandbox-derived numerical features encode temporal statistics such as connection frequency, entropy, and fan-out into aggregated form.

**Figure 10:**
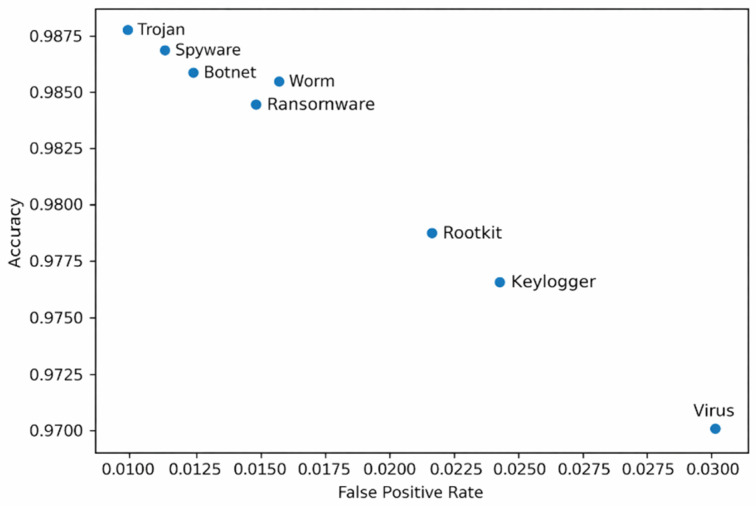
Accuracy versus FPR trade-off for the best model in each malware category.

LightGBM efficiently exploits these threshold-based indicators using gradient-based one-side sampling and leaf-wise tree growth, achieving strong discrimination without requiring explicit temporal sequence modeling. This explains why recurrent and convolutional deep models do not offer a performance advantage in this setting. Ransomware is the only malware category for which a deep learning model, TabNet, emerges as the top performer. TabNet’s sequential attention mechanism enables dynamic feature selection at the sample level, allowing the model to focus on a small subset of highly informative features while suppressing irrelevant background activity. This behavior-aligned inductive bias explains why TabNet surpasses even strong tree-based ensembles for ransomware detection. Rootkit detection favors the Extra Trees classifier, as illustrated by its strong performance in Figure 8. The randomized split selection and ensemble averaging employed by Extra Trees reduce variance and mitigate sensitivity to noise, leading to improved generalization and lower false-positive rates. In contrast, deep learning models tend to overfit minor fluctuations in such low-signal regimes, resulting in inferior performance. For worm malware, XGBoost achieves the best results, driven by its ability to model propagation-oriented behavior such as scanning, replication, and rapid process spawning. XGBoost’s regularized gradient boosting framework effectively captures threshold-dependent relationships while maintaining stability across feature interactions, making it particularly well suited for modeling worm propagation dynamics. Spyware detection again favors CatBoost, underscoring its strength in modeling stealthy malware that closely resembles benign software. Keylogger detection is best addressed by Random Forests. Ensemble averaging allows Random Forests to aggregate these weak signals effectively, providing stable detection performance without excessive sensitivity to feature redundancy. As shown in Figure 8, the FT-Transformer performs best in this category by leveraging feature embeddings and attention mechanisms to model cross-feature dependencies between static attributes and runtime behavior. The virus category also exhibits a higher false-positive rate compared with tree-based models, indicating that while tabular deep learning can be competitive, it does not universally outperform ensemble methods. The trade-off between detection accuracy and false-positive rate across malware categories is further visualized in Figure 10. Ensemble tree models consistently occupy favorable regions of this trade-off space, reinforcing their suitability for practical IoT and deployment.

**Figure 11:**
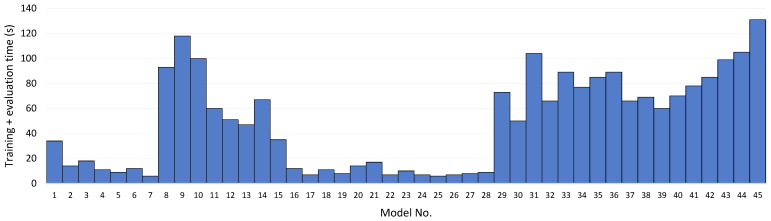
Computational time analysis of all 45 evaluated models.

To obtain a more in-depth understanding of the practical deployability of the evaluated approaches and to provide actionable recommendations beyond predictive performance, a computational time analysis was conducted across all 45 models under a consistent training and evaluation protocol on the generated IoT malware dataset. Figure 11 reports the end-to-end wall-clock cost for each model, indexed by the same model numbering used in Table 4. Experiments were conducted on a Dell OptiPlex 7780 AIO workstation (Dell Technologies, Round Rock, TX, USA) running Windows 11 Enterprise, equipped with an Intel Core i7-10700 CPU (8 cores, 16 threads, 2.90 GHz) and 32 GB RAM. All models were trained and evaluated using the same fixed train–test split and software configuration. Classical ML models used their standard implementations, while deep learning models were trained with a fixed batch size of 128, a maximum of 50 epochs, and early stopping based on validation loss. For tree-based ensembles (CatBoost, LightGBM, and XGBoost), hyperparameters including learning rate, tree depth, and number of estimators were optimized using a randomized grid search across all iterations.

The results reveal a clear separation between classical ML methods and several deep learning families in terms of computational efficiency. Linear and probabilistic baselines (e.g., Logistic Regression, Ridge, LDA/QDA, Naïve Bayes, and SGD-type classifiers) exhibit the lowest runtime due to closed-form or near-linear optimization and relatively small parameter spaces, making them suitable for rapid iteration and resource-constrained environments, albeit at a moderate performance cost. Among the top-performing models, gradient-boosted tree ensembles (LightGBM, XGBoost, CatBoost) demonstrate a favorable accuracy–time trade-off: while their training cost is higher than simple linear models, they remain substantially more efficient than most deep architectures while achieving superior detection performance. CatBoost typically incurs higher overhead than LightGBM and XGBoost due to its boosting strategy and implementation details, but it still remains in a practical regime compared with heavier deep models.

In contrast, deep learning methods that ranked highly in Table 4, particularly TabNet and Transformer-based models (Transformer Encoder and FT-Transformer), incur noticeably higher computational cost. This is consistent with their reliance on iterative gradient-based training over larger parameterized architectures, attention mechanisms, and multiple training epochs, all of which impose a higher constant factor even when the input dimensionality is modest. Deep sequential models also exhibit elevated runtime because they introduce architectural priors that do not map efficiently to a compact, engineered feature vector; consequently, they pay additional computational cost without a commensurate accuracy gain relative to tree ensembles. The most computationally expensive group includes generative families which typically require more complex optimization dynamics, additional sampling steps, or message-passing computations; these models also underperform in Table 4, indicating an unfavorable accuracy–time profile for the present tabular telemetry formulation. The time analysis supports the primary performance-based conclusion.

## 6. Recommendations

Based on the comprehensive performance evaluation, per-malware analysis, and computational time assessment presented in this study, several evidence-driven recommendations can be made for the practical deployment of IoT malware detection systems. These recommendations are derived from systematic experimentation on a large-scale, feature-engineered numerical dataset that closely mimics realistic IoT and IIoT operational environments, while acknowledging that no single configuration can be universally optimal across all deployment contexts. For general-purpose IoT malware detection where heterogeneous threats are expected and computational efficiency is a primary constraint, ensemble tree-based machine learning models particularly gradient-boosted trees should be adopted as the default backbone. Models such as CatBoost, LightGBM, and XGBoost consistently achieve near-ceiling detection accuracy, high F1-scores, and low false-positive rates across most malware categories while maintaining favorable runtime characteristics on commodity CPU hardware. Their effectiveness is strongly tied to the nature of the dataset used in this study. In such tabular settings, ensemble trees efficiently model high-order, non-linear feature interactions without incurring the computational overhead associated with deep representation learning. Consequently, these models provide the strongest accuracy–runtime trade-off for realistic IoT and IIoT environments that rely on aggregated telemetry from diverse sensors and system logs. For Trojan and Spyware detection, CatBoost is specifically recommended. The results demonstrate that CatBoost achieves the highest accuracy and lowest false-positive rates for these malware types. Its ordered boosting strategy and regularization mechanisms enable reliable generalization under class imbalance, making it well suited for IoT deployments where false alarms can disrupt system operation or erode trust in security mechanisms. For Botnet and Worm detection, LightGBM and XGBoost, respectively, are recommended. These malware classes are dominated by network-centric and propagation-oriented behaviors, explaining why recurrent and convolutional deep learning models do not offer additional benefits in this context. Given their relatively low computational cost and strong recall performance, these models are well suited for real-time detection of threats in IoT networks. For Rootkit and Keylogger detection, Extra Trees and Random Forests are recommended. Their ensemble averaging and randomized splitting strategies reduce variance and mitigate sensitivity to noise, leading to improved stability and lower false-positive rates. These characteristics are particularly valuable for detecting stealthy malware that intentionally avoids triggering strong behavioral signatures. For ransomware detection, this study recommends the use of TabNet, as it is the only category in which a deep learning model consistently outperforms ensemble ML alternatives. TabNet’s sequential attention-based feature selection allows it to dynamically focus on these highly informative subsets at inference time, providing a measurable advantage over tree-based methods. However, due to its higher computational cost, TabNet is best deployed selectively either as a specialized detector for ransomware-prone environments or as part of a hybrid system triggered by preliminary screening. For Virus detection, FT-Transformer demonstrates the strongest performance. Its attention-based feature embedding mechanism enables effective modeling of cross-feature dependencies. Nevertheless, its comparatively higher false-positive rate and computational overhead indicate that it should be deployed with caution and primarily in environments where virus-like threats are prevalent and sufficient computational resources are available.

Across all malware categories, the computational time analysis reinforces these recommendations by demonstrating that ensemble ML methods deliver consistently high detection performance at substantially lower runtime cost than most deep learning architectures. Deep sequential and generative models not only incur significantly higher computational overhead but also underperform in accuracy on the present dataset, indicating an unfavorable accuracy–time profile for feature-engineered tabular telemetry. As such, these models are not recommended for direct deployment in similar IoT settings without a fundamental redesign of the data representation that includes event sequences or graph structures. It is important to emphasize that these recommendations are data-dependent. The dataset used in this study is designed to closely reflect a realistic IoT threat environment by combining static and behavioral features extracted from sandbox execution. While this formulation is representative of many contemporary IoT and IIoT deployments, particularly those relying on aggregated numerical monitoring data, the conclusions should not be assumed to generalize to all scenarios. Environments that provide raw temporal traces, packet-level streams, or explicit relational graphs may benefit from alternative modeling paradigms. Nevertheless, for a wide range of practical IoT systems that utilize heterogeneous sensors and summarized behavioral features, the presented results offer robust, actionable guidance.

## 7. Conclusions

The widespread adoption of Internet-of-Things (IoT) technologies across modern enterprises and industrial systems has significantly increased the exposure of cyber–physical infrastructures to malware threats. As most operational environments now rely on internet-connected sensors and devices, ensuring robust and scalable malware detection has become essential for maintaining system safety and reliability. Achieving this objective requires a comprehensive detection pipeline, encompassing realistic data acquisition, careful feature engineering, and rigorous model evaluation. This study emphasizes that effective IoT malware detection begins with the construction of a reliable, large-scale dataset that reflects real-world threat behavior, followed by careful feature extraction and selection that preserves discriminative information while avoiding unnecessary complexity. By generating a numerical dataset of 50,000 samples derived from sandboxed malware execution and behavioral analysis, and by reducing the feature space to 47 highly informative attributes, this work establishes a realistic and practical foundation for model evaluation. Such a formulation mirrors the type of aggregated telemetry commonly available in enterprise and industrial IoT environments, where raw packet traces or event streams are often summarized into numerical indicators. To obtain a holistic view of detection capability, this study conducted one of the most extensive comparative evaluations to date, benchmarking 27 machine learning models and 18 deep learning architectures across eight major malware categories. The results consistently demonstrate that classical machine learning approaches—particularly ensemble and gradient-boosted tree methods—provide superior performance in this setting. Notably, seven of the top-ten-performing models are machine learning models, underscoring their effectiveness in modeling engineered tabular features. These models achieve high accuracy, precision, recall, and F1-scores while maintaining low false-positive rates and favorable computational efficiency. The findings further reveal that deep learning models become competitive only when their architectural assumptions align closely with the structure of the data, as observed for ransomware and virus detection using tabular attention-based models. However, for the majority of malware types and for general-purpose deployment, deep architectures incur higher computational cost without delivering commensurate performance gains. This observation is reinforced by the computational time analysis, which highlights a clear separation between ensemble ML models and most deep learning families in terms of runtime feasibility on commodity hardware.

## Figures and Tables

**Table 1:** IoT malware detection approaches and their applicability across attack scenarios and system conditions.

	Attack Mechanism							Attack Type			Attack Layer				Delivery Mechanism					Attack Objective					Attack Size			Security Void			
	Resource Exhaustion	Network Traffic	Unauthorized Access	Data Integrity	Reconnaissance Behavior	Power-State Manipulation	Volumetric Based	Protocol Misuse	Behavior Anomalies	Sensing-Level	Edge/Gateway Layer	Network Layer	Application Layer	Packet-Level Traffic	Malicious Payload	Botnet Control Artifacts	Exploit Behavior	Credential Access	Bandwidth	Resource Abuse	Device Depletion	Data Exfiltration	Device Hijacking	Bit-Rate Anomalies	Packet-Rate	Request-Rate	Insecure Channels	Weak Authentication	Outdated Firmware	Software Exploitability	Data Handling
Signature-Based			x						x				x							x	x	x									x
Polymorphic Malware		x	x		x		x	x			x			x		x		x				x	x					x			
Behavior-Based		x	x					x					x					x	x	x			x				x	x			
Sandbox Analysis					x	x		x					x		x	x				x		x									
Heuristic-Based Detection	x								x				x							x						x					
Model Checking-Based		x				x		x				x					x				x				x		x		x		
ML-Based Detection		x		x		x			x		x		x			x		x					x			x		x			
ML (Feature-Based)				x					x	x			x		x					x						x				x	
DL-Based Detection				x				x					x			x						x									
DL (Sequence)	x						x					x		x				x	x					x			x				
Cloud-Based	x						x		x		x	x		x		x			x					x			x				
Cloud-Assisted IoT				x					x				x					x				x									x
IoT-Specific Malware			x						x				x				x			x						x				x	
IoT Edge-Level				x		x			x				x		x			x		x						x				x	
IoT Malware Datasets		x					x			x				x		x			x					x							
Proposed Dataset Framework				x					x				x		x					x						x				x	
Proposed Feature-Selected	x							x				x						x				x				x				x	
Physical Node Anomalies			x					x		x							x				x				x				x		
Passive Anomalies					x			x					x									x					x				
Social Engineering				x				x				x						x					x								x
Malformed Packet		x					x			x		x		x					x					x							
Data Encryption									x				x								x										
Unauthorized Firmware			x						x				x								x									x	
Routing Anomalies		x						x			x						x			x					x					x	x
Service Disruption Anomalies	x						x		x	x			x							x					x						x
Session Violation Anomalies				x					x				x										x								
Side-Channel Anomalies				x					x				x									x									
Power Consumption						x		x		x											x				x		x				
Traffic Amplification		x					x					x		x					x					x			x				
Traffic Monitoring				x					x				x					x				x									x
Storage Abuse									x		x										x					x					x
Fragmentation		x						x					x	x					x					x						x	
Pattern Inference				x					x	x		x										x									
Enumeration					x			x					x				x					x								x	
Wireless					x			x				x										x									
Stealth Persistence			x						x			x				x					x								x		

**Table 2:** Selected diagnostic features used for dataset construction.

Feature Category	Feature Name	Feature Category	Feature Name
Static	File size		API call frequency
	PE file entropy	File	Number of files created
	Compiler value		Number of files modified
	Imported library count		File deletion count
Behavioral	Suspicious behavior attempt		Access to system directories
	Persistence behavior attempt		Creation of hidden files
	Privilege escalation attempt		File write frequency
	Evasion behavior value	Registry	Number of registry keys created
	Process injection value		Number of registry keys modified
	Self-modifying code value		Access to autorun registry entries
	Delayed execution value		Registry persistence value
	Unauthorized system flag		Registry access frequency
Configuration	Execution environment value		Unauthorized registry access
	Use of elevated privileges	Debug	Debugger detection value
	Sandbox detection value		Use of anti-debugging APIs
	Variable modification		Exception handling anomalies
	System configuration attempt		Timing-based evasion value
Process	Number of processes created	Network	Number of outbound connections
	Suspicious parent–child process		Destination IP diversity
	Process memory usage peak		Unusual port usage
	Process injection attempt		Network packet rate
	Process termination anomalies		Data volume transmitted
	Hidden process creation		Encrypted communication attempt
	Command-line entropy		

**Table 3:** List of ML and DL models evaluated on the generated IoT malware dataset.

No.	ML Models	No.	DL Models
1	Logistic Regression	1	Multilayer Perceptron (MLP)
2	Naïve Bayes (Gaussian)	2	Deep Neural Network (DNN)
3	Naïve Bayes (Multinomial)	3	Residual MLP
4	Linear Discriminant Analysis	4	1D Convolutional Neural Network
5	Quadratic Discriminant Analysis	5	Autoencoder (AE)
6	Ridge Classifier	6	Stacked Autoencoder (SAE)
7	Support Vector Machine (Linear)	7	Variational Autoencoder (VAE)
8	Support Vector Machine (RBF)	8	Denoising Autoencoder
9	k-Nearest Neighbors	9	Transformer Encoder
10	Decision Tree	10	TabNet
11	Random Forest	11	FT-Transformer
12	Extra Trees	12	Generative Adversarial Network (GAN)
13	Gradient Boosting	13	CTGAN
14	AdaBoost	14	Deep Belief Network (DBN)
15	XGBoost	15	Deep Boltzmann Machine (DBM)
16	LightGBM	16	Capsule Network
17	CatBoost	17	Attention-based Neural Network
18	SGD Classifier	18	Multi-task Neural Network
19	Passive-Aggressive Classifier		
20	Perceptron		
21	Extreme Learning Machine		
22	One-Class SVM		
23	Isolation Forest		
24	Local Outlier Factor		
25	Elliptic Envelope		
26	Bayesian Network		
27	RuleFit		

**Table 4:** Performance comparison of ML and DL models on the generated IoT malware dataset.

No.	Model	Model Name	Train Acc	Acc	Precision	Recall	F1	FPR
1	ML	CatBoost	0.991	0.990	0.990	0.979	0.989	0.008
2	ML	LightGBM	0.990	0.987	0.979	0.989	0.984	0.011
3	ML	XGBoost	0.989	0.984	0.987	0.985	0.986	0.014
4	ML	Gradient Boosting	0.987	0.980	0.980	0.973	0.977	0.017
5	ML	Extra Trees	0.987	0.976	0.975	0.974	0.977	0.020
6	ML	Random Forest	0.986	0.974	0.972	0.973	0.973	0.023
7	ML	Support Vector Machine (RBF)	0.984	0.971	0.970	0.969	0.970	0.026
8	DL	TabNet	0.985	0.968	0.970	0.965	0.967	0.029
9	DL	FT-Transformer	0.984	0.965	0.963	0.960	0.962	0.032
10	DL	Transformer Encoder	0.981	0.962	0.965	0.958	0.961	0.035
11	DL	Attention-based NN Network	0.981	0.958	0.956	0.960	0.958	0.038
12	DL	Deep Neural Network (DNN)	0.979	0.955	0.954	0.951	0.952	0.041
13	DL	Residual MLP	0.976	0.952	0.950	0.951	0.951	0.044
14	DL	Multilayer Perceptron (MLP)	0.973	0.949	0.952	0.944	0.948	0.047
15	ML	RuleFit	0.960	0.946	0.949	0.945	0.947	0.050
16	ML	Linear Discriminant Analysis	0.956	0.942	0.943	0.940	0.942	0.053
17	ML	Ridge Classifier	0.953	0.939	0.936	0.943	0.939	0.056
18	ML	Support Vector Machine	0.950	0.936	0.938	0.932	0.935	0.059
19	ML	Logistic Regression	0.947	0.933	0.933	0.927	0.930	0.062
20	ML	Quadratic Discriminant Analysis	0.944	0.929	0.928	0.931	0.929	0.065
21	ML	k-Nearest Neighbors (kNN)	0.943	0.926	0.930	0.926	0.928	0.068
22	ML	Decision Tree	0.953	0.923	0.922	0.919	0.920	0.071
23	ML	AdaBoost	0.950	0.920	0.915	0.921	0.918	0.074
24	ML	SGD Classifier	0.936	0.917	0.918	0.914	0.916	0.077
25	ML	Passive-Aggressive Classifier	0.924	0.914	0.915	0.912	0.914	0.080
26	ML	Perceptron	0.921	0.910	0.907	0.912	0.909	0.083
27	ML	Naïve Bayes (Gaussian)	0.915	0.907	0.910	0.901	0.905	0.086
28	ML	Naïve Bayes (Multinomial)	0.913	0.904	0.901	0.906	0.903	0.089
29	DL	Multi-task Neural Network	0.890	0.869	0.868	0.870	0.869	0.122
30	DL	Autoencoder (AE)	0.892	0.866	0.868	0.862	0.865	0.125
31	DL	Denoising Autoencoder	0.879	0.863	0.860	0.865	0.862	0.128
32	DL	Stacked Autoencoder (SAE)	0.877	0.859	0.860	0.857	0.858	0.131
33	DL	Variational Autoencoder	0.855	0.856	0.858	0.853	0.855	0.134
34	ML	Isolation Forest	0.844	0.853	0.851	0.856	0.853	0.137
35	ML	One-Class SVM	0.831	0.850	0.854	0.848	0.851	0.140
36	ML	Local Outlier Factor (LOF)	0.839	0.847	0.845	0.849	0.847	0.143
37	ML	Elliptic Envelope	0.827	0.843	0.845	0.840	0.842	0.146
38	ML	Extreme Learning Machine	0.825	0.840	0.836	0.844	0.840	0.149
39	ML	Bayesian Network	0.821	0.837	0.838	0.835	0.836	0.152
40	DL	Capsule Network	0.817	0.834	0.835	0.831	0.833	0.155
41	DL	Deep Belief Network (DBN)	0.815	0.831	0.833	0.827	0.830	0.158
42	DL	Deep Boltzmann Machine	0.812	0.828	0.829	0.825	0.827	0.161
43	DL	GAN	0.808	0.825	0.829	0.834	0.831	0.168
44	DL	CTGAN	0.803	0.822	0.826	0.827	0.826	0.171
45	DL	1D CNN	0.801	0.810	0.812	0.815	0.809	0.177

**Table 5:** Best-performing model per malware category.

Malware	Best Model	Model	Train Acc	Acc	Precision	Recall	F1	FPR
Trojan	CatBoost	ML	0.992	0.988	0.989	0.977	0.983	0.010
Botnet	LightGBM	ML	0.989	0.986	0.984	0.988	0.986	0.012
Ransomware	TabNet	DL	0.983	0.984	0.983	0.981	0.982	0.014
Rootkit	Extra Trees	ML	0.987	0.978	0.976	0.974	0.975	0.020
Worm	XGBoost	ML	0.991	0.985	0.986	0.984	0.985	0.015
Spyware	CatBoost	ML	0.990	0.987	0.988	0.976	0.982	0.011
Keylogger	Random Forest	ML	0.986	0.976	0.975	0.972	0.973	0.022
Virus	FT-Transformer	DL	0.985	0.969	0.968	0.965	0.966	0.030

## Data Availability

The datasets generated and analyzed during the current study are available from the first author.
